# Experience of an English Dentist Who Had Two Teeth Implanted

**Published:** 1887-12

**Authors:** J. H. Gartrell


### Editorial, Etc.

---

THE following letter, from the London *Dental Record*, gives the experience of an English dentist who had two teeth implanted by Dr. Younger at a clinic held in Washington, D. C., during the session of the International Medical Congress.

PENZANCE, October 26th, 1887.

DEAR CUNNINGHAM.—In reply to your enquiry *in re* notes on my implantation case, I send you the two teeth used, and I shall be glad if you will have them examined under a microscope, and let me know if there is any pericemental membrane on them or not. They are, as you see, the right and left superior laterals, but whence they came, and to whom they belonged, I cannot say. I believe they were picked out from a lot of extracted teeth at some dentist's in Washington. Nearly all who spoke to me as to the operation were curious to know the severity as regards pain. My answer has been that it is quite as bad as having a tooth out. After the first incision the detaching of the periosteum, before drilling the hole, is the most painful. The drilling and burring out of the socket is, however, bad enough, and got worse as it neared the end. In my case, however, I think it was worse than usual, as the whole of the outer, or labial, plate of the socket for the right lateral, was cut away; and the instrument finally passed through the gum on the labial surface, which was afterwards held together by a stitch of silk thread. The left lateral had a very thin plate of bone on its labial surface, but the trouble with this tooth was that it was a very thick one, as you can see by looking at it, so that it came in the way of the bite. Dr. Younger tried to correct this by grinding off the cutting edges of the lower laterals, but he did not take off enough to do any good; the consequence was that after five or six days' use the silk ligatures had slackened a little, the teeth got very loose and moved with the least possible force—such as suction in drinking, &c. The right lateral could also with very slight pressure be pushed up into the gum an eighth of an inch too far, the root pressing the gum outwards before it. The left lateral was also being constantly interfered with by the lower teeth; also the hurry sometimes in travelling prevented my taking as much care of them as I might have done at home. In fact, there ought to have been a platinum plate struck up to fit the palatal surfaces of the whole of the front teeth, with the platinum passing up over the cutting edges of the two implanted teeth, so as to protect them and to keep them steadily in their place. I got my cousin, a dentist in Ottawa, in Canada, to tie the teeth with fresh ligatures, but they got so loose and uncomfortable that I concluded that it was useless to persevere any longer; but I will try the operation again at home when I can get two fresh good teeth and can give proper attention to taking care of them afterwards. In my case Dr. Younger worked very hurriedly, taking about six minutes in inserting the left lateral, reckoning from the first incision to the tooth being placed *in situ*. I felt that I could have stood the pain better if a longer time was taken, but he wished to operate against time and astonish the natives. I think he made a mistake in not leaving a plate of bone on the labial surface of the right socket by drilling the hole more towards the palate. This might perhaps have interfered with the tooth taking its place in the arch properly; in which case I think it would be better to place an artificial tooth upon a natural root at the proper angle. I should have been glad if cocaine had been tried in drilling one of the sockets, so as to note the difference in the pain, but the whole operation was done hurriedly, and proper care was not taken afterwards, which no doubt accounts for its failure.—Yours sincerely, J. H. GARTRELL.

